# The association between herpes virus infections and functional somatic symptoms in a general population of adolescents. The TRAILS study

**DOI:** 10.1371/journal.pone.0185608

**Published:** 2017-10-18

**Authors:** Iris Jonker, Robert Schoevers, Hans Klein, Judith Rosmalen

**Affiliations:** 1 University of Groningen, University Medical Center Groningen, Department of Psychiatry, Interdisciplinary Center for Psychopathology and Emotion Regulation (ICPE), Groningen, The Netherlands; 2 Department of Internal Medicine, University of Groningen, University Medical Centre Groningen, Groningen, The Netherlands; University of St Andrews, UNITED KINGDOM

## Abstract

**Background:**

FSS have been suggested to follow activation of the immune system, triggered by herpes virus infections. The aim of this study was to find out whether herpes virus infections were associated with the experience of FSS in adolescents, and whether this association was mediated by hsCRP, as a general marker of immune activation.

**Methods:**

This study was performed in TRAILS, a large prospective population cohort of 2230 adolescents (mean age: 16.1 years, SD = .66, 53.4% girls). FSS were assessed using the somatic complaints subscale of the Youth Self-Report. FSS were analyzed as total scores and divided in two group clusters based on previous studies in this cohort. Levels of hsCRP and antibody levels to the herpes viruses HSV1, HSV2, CMV, EBV and HHV6 were assessed in blood samples at age 16. Also a value for pathogen burden was created adding the number of viruses the adolescents were seropositive for. Multiple regression analysis with bootstrapping was used to analyze the association between viral antibodies and pathogen burden, hsCRP and FSS scores.

**Results:**

Antibody levels and pathogen burden were not associated with FSS total scores or FSS scores in both symptom groups. hsCRP was associated with the total FSS score (B = .02, 95% CI: .004 to .028, p = .01) and FSS score in the symptom group of headache and gastrointestinal complaints (B = .02, 95% CI: .001 to .039, p = .04).

**Conclusion:**

Our study showed no association between herpes virus infections and FSS in general or specific FSS symptom clusters. A role for inflammatory processes in FSS development was supported by the significant association we found between hsCRP levels and FSS, especially in the symptom group of headache and gastrointestinal complaints.

## Introduction

Functional somatic symptoms (FSS) are symptoms for which no conclusive medical explanation can be found. FSS are common in children and adolescents and lead to school absence and frequent medical attention seeking. The etiology of FSS is largely unknown. Psychological, social and biological factors are believed to contribute to FSS [1]. These factors can have predisposing, precipitating and perpetuating influences in the etiology of FSS. One of the biological factors suggested to play a role in FSS is low grade activation of the immune system [2]. Viral infections have been suggested to be a precipitating factor in the development of FSS and could lead to such low-grade inflammation [3]. Particularly herpes virus infections are interesting in this regard, since they have the ability to be latent in the body and are reactivated from time to time, leading to a state of chronic immune activation [4]. High viral antibody levels may represent recent viral activity, which in turn points to an impairment of the immune system, as it is not able to prevent subtle reactivations of the virus [5]. Only one study focused on herpes virus infections as a precipitating factor in FSS. It was found that viral antibody levels against EBV were associated with FSS scores in an adult population [6].

Not many studies focused on the role of inflammation and infection in FSS, but there have been studies on the role of inflammation and infection in functional somatic syndromes. Functional somatic syndromes are clusters of FSS, and include Chronic Fatigue Syndrome (CFS), Fibromyalgia Syndrome (FMS) and Irritable Bowel Syndrome (IBS). Several studies showed that infections might precipitate development of these syndromes [7,8]. High prevalences of antibodies to Human Herpes Virus 6 (HHV6) [9] and Epstein Barr Virus (EBV) [10] were reported in CFS patients, but results remain inconsistent [11–14] and associations appear absent in FMS [14]. Onset of irritable bowel syndrome was found to be associated with infectious gastroenteritis [15] and with chronic Hepatitis C infection [16]. In summary, several links have been suggested between a variety of viral infections and a diverse set of functional somatic syndromes, but results are equivocal.

Several questions remain considering the association between FSS and herpes infections. The first question that arises is whether viral infection in general might be associated with specific types of FSS. In one study it was found that EBV infection was a risk factor for developing CFS, whereas campylobacter gastroenteritis was a risk factor for developing IBS [17]. This might suggest that viral infections are more related to general symptoms such as pain or fatigue than to gastrointestinal complaints. The second question is whether FSS might be particularly associated with specific viruses. Previous studies have also suggested that it is not the type of viral infection that confers the risk of FSS, but rather the number of different viral infections, referred to as viral load or pathogen burden. Supporting this idea, a registry-based study revealed that patients with multiple FSS had a significantly higher infection load in the period preceding their symptoms than controls [18]. Thus, pathogen burden might be more important than the exact type of viral infection. An important third question is whether viral infection leads to low-grade inflammation, which in turn leads to FSS. Previous studies have suggested that FSS might be associated with low-grade inflammation, with C-reactive protein (CRP) or pro-inflammatory cytokines as indicators of this low-grade inflammation [2,19]. The cause of this low grade inflammation remains unknown. The vicious circle leading to FSS may be precipitated by viral infection. When FSS become chronic, perpetuating factors such as cognitive processes may become more important. Adolescence is an interesting age to study the precipitating factors that initiate this vicious circle, since at this age the perpetuating factors may be less important.

The aim of this study is to find out whether antibody levels against different herpes viruses are associated with FSS. Our first hypothesis is that the associations with viral antibody levels are particularly present for fatigue and pain symptoms, and less prominent for gastrointestinal symptoms. Our second hypothesis is that pathogen burden is more strongly associated with FSS than the exact type of viral infection. We will also test whether there is a role for CRP as a marker of systemic inflammation in this association between viral infection and FSS.

In the adolescent cohort “TRacking Adolescents’ Individual Lives Survey” (TRAILS), we will study the association between FSS, low grade immune activation and herpes virus infections at age 16.

## Methods

### Study population

The study is part of the TRacking Adolescents’ Individual Lives Survey (TRAILS), which is a large prospective population study of 2230 Dutch adolescents from the north of the Netherlands examining causes and outcome of physical and mental health from childhood into adulthood. The survey was approved by the national ethical committee “Centrale Commissie Mensgebonden Onderzoek”. Written Informed Consent was obtained from children and their parents or caretakers. A more elaborate description of the design can be found elsewhere [20]. In short, all 135 primary schools in the five major municipalities in the northern part of the Netherlands were asked to participate in this study. Of the children of the schools that decided to participate, subjects with mental retardation, physical incapability or language problems were excluded. The study started in 2001 with the first wave of measurements, after that three follow up measurements were done every two years. During the third assessment, all participants were asked to donate a blood sample for different biomarker analyses. After consent had been obtained from the parents and the children, blood samples were taken at the schools and delivered to the University Medical Center Groningen, where serum was extracted and stored at −80°C until analysis. We had data on inflammatory markers and FSS scores in a population of 1220 participants.

### Functional somatic symptoms

The Somatic Complaints Subscale of the Youth Self Report was used to estimate the amount of FSS [21]. This questionnaire is known to have a good cross-cultural validity [22]. The Somatic Complaints Subscale consists of 9 items that refer to somatic complaints without a medical cause or without any apparent reason. The adolescents could indicate whether they experienced these complaints on a three-point scale with response categories “not at all”, “sometimes” and “often”. Since the Youth Self-Report did not include musculoskeletal symptoms, those symptoms were assessed by asking participants questions about how often they experienced pain in their neck, back, shoulders, arms and legs during the past three months. Questions were rated on a 7-point measurement scale with response categories: ‘Not at all’, ‘Less than once a month’, ‘Once a month’, ‘Two to three times a month’, ‘Once a week’, ‘Two to six times a week’, and ‘Almost every day’. A mean item score of the three gastrointestinal symptoms and of the five musculoskeletal symptoms was created. The mean item score of the five musculoskeletal pain symptoms was divided by three-and-a-half to rescale to the YSR. An exploratory factor analysis was performed that supported the division of symptoms into two clusters, one consisting of headache and gastrointestinal complaints, and the other consisting of overtiredness, dizziness and musculoskeletal pain. This factor analysis process was described in more detail in a previous article in the same study population [23]. For the FSS total scores we combined results of both questionnaires.

### Measurement of antibodies

All serological tests were carried out by standard procedure at the Stanley Laboratory of Developmental Neurovirology, Baltimore, Maryland. To estimate viral activity, IgG serum antibodies against agents of the herpes virus family (HSV1, HSV2, EBV, CMV and HHV) were measured. For this measurement a modified solid-enzym immunoassay method was used, as was described before [24]. The immunoassays were performed by the reaction of the serum samples against the solid-phase antigen. To differentiate between the antibodies of HSV-1 and HSV-2, purified viral-envelope glycoproteins were used as the solid-phase agents, namely gG1 for HSV1 en gG2 for HSV2 [25]. Reagents for these assays were obtained from Focus Laboratories, Inc (Cypress, Calif). The assays for the antibodies to HHV, CMV and EBV used antigens derived from virion proteins as previously described [24,26]. Reagents for these assays were obtained from IBL-Hamburg GmbH (Hamburg, Germany). After rinsing the serum samples that did not react to the antigens, the amount of antibody that was bound to the solid-phase antigen was quantified, by letting it react with enzyme labeled anti-human IgG and enzyme substrate. This process created a visual colored substrate, of which the optical density could be measured in a microplate colorimeter. Every assay was performed with our participant sample and a reference sample with low but detectable levels of antibodies against the target antigen. The amount of antibody in every sample was expressed in terms of the ratio of optical density of the test sample against that of the standard sample and was expressed in arbitrary units (AU). For the first estimation of positivity, the sample of a participant was labeled as reactive when the ratio was at least 1.1. This cut-off point was based on a clinical trial of the assays of antibodies against HSV-1 and HSV-2 [27]. For EBV and CMV positivity also a ratio of 1.1 was used, for HHV positivity a ratio of 2.0 was used [24]. As a sensitivity analysis, we performed additional analyses with the continuous antibody levels in the total group, and with antibody levels in the seropositive group using ratios of 0.5 and 2.0 to define reactivity. This did not yield different results. Pathogen burden was defined as the number of herpes viruses the participants had antibodies to.

### Measurement of hsCRP

CRP is produced by the liver in reaction to pro-inflammatory cytokines and is a measurement for systemic inflammation. High sensitive-CRP (hsCRP) is a measurement of CRP that is more sensitive to small changes in systemic inflammation, since it is detected at lower values [28]. hsCRP was determined using an immunonephelometric method, BN2, with a lower detection limit of 0.175 mg/L. Intra-assay coefficients of variance ranged from 2.1 to 4.4, and inter-assay coefficients of variation coefficients of variance ranged from 1.1 to 4.0. In our sample, hsCRP values were relatively low, as could be expected from a general sample of adolescents. Median value was 0.4 mg/L, with 25th and 75th percentiles respectively 0.2 and 1.0 mg/L. Using continuous values of hsCRP would imply a floor effect with a risk of false negative results. For this reason we decided to repeat analyses with dichotomized hsCRP values, with elevated hsCRP indicated by a cut-off of 2.0 mg/L. This cutoff was chosen in a previous study in the same cohort, based on studies on inflammation in mood disorders [29–31]. For consistency between studies we decided to use the same cutoff in this study. Participants with hsCRP > 10 mg/L were excluded from the analyses (n = 33).

### Confounders

Several potential confounders exist in the relationship between inflammation and the experience of FSS. Female sex [32–34], high BMI [32,35] (, and affective symptoms [36,37] are all related to both inflammatory markers and FSS. Therefore we adjusted all our analyses for these potential confounders. Length and weight of the adolescents were measured to calculate BMI. Affective symptoms were assessed using the Affective Problems scale of the Youth Self-Report (13 items, Cronbach's alpha = .75) [38].

### Statistical analysis

To test our first hypothesis on the association between FSS in different symptom groups and viral antibody levels, we performed multiple linear regression analysis, with the antibody levels as predictor and FSS scores as dependent factor. This was done with FSS total score and with FSS scores in the two symptom clusters, in the seropositive group. Given that only 13 adolescents were HSV2-positive, we did not perform regression analyses for this virus. To test our second hypothesis that pathogen burden was associated with the FSS scores, we repeated these analyses with pathogen burden as predictor and FSS scores as dependent factor. To test our third hypothesis that viral antibodies lead to FSS via low grade inflammation, we first tested whether the hsCRP levels were associated with FSS scores, using multiple regression analysis, with hsCRP levels as predictor and FSS scores as dependent factor. We then tested whether the amount of viral antibodies in the seropositive group was associated with hsCRP levels, also performing regression analyses with viral antibodies as predictor and hsCRP as dependent factor. In case of an association between viral antibodies and hsCRP, we planned to repeat the regression analyses with CRP added in the model as mediator. Because of non-normal distribution of data, we performed bootstrapping analysis in our regression models. Bootstrapping gets around the problem of non-normal distribution by estimating the properties of the sampling distribution from sample data (called bootstrapping samples). The values of the samples are used to estimate the limits of the 95% confidence interval of the parameter. An effect is considered significant when the bootstrap 95%-CI does not include zero [39]. All analyses were adjusted for sex, affective symptom scores, and BMI. The analyses with FSS symptom clusters as outcome were additionally adjusted for scores in the other symptom cluster.

## Results

### General characteristics

The population consisted of 1220 adolescents; the population characteristics are summarized in Table 1.

**Table 1:** Population characteristics.

General Characteristics	N (%)	Median (25^th^, 75^th^ percentile), unless otherwise specified
Age (years), Mean (SD)		16.1 (0.66)
Female sex	651 (53.4)	
Dutch Ethnicity	1093 (89.6)	
BMI (kg/m2)		20.8 (19.2, 22.6)
hsCRP (mg/l)		0.4 (0.2, 1.0)
Elevated CRP (>2mg/L)	154 (13)	
**Antibody status:** **Number of adolescents with antibodies and median levels of antibodies (AU)**		
HSV1	301 (24.7)	2.09 (1.68, 2.53)^#^
HSV2	13 (1.1)	1.67 (1.56, 2.12)^#^
EBV	305 (25.0)	1.30 (1.19, 1.49)^#^
CMV	306 (25.1)	2.19 (1.95, 2.52)^#^
HHV6	1117 (91.6)	5.01 (3.88, 7.11)^#^
**Pathogen Burden, number of viruses that the participants had antibodies to**		
Antibodies to none of the pathogens	47 (3.9)	
Antibodies to 1 pathogen	535 (43.9)	
Antibodies to 2 pathogens	446 (36.6)	
Antibodies to 3 pathogens	154 (12.6)	
Antibodies to 4 pathogens	37 (3.0)	
Antibodies to 5 pathogens	1 (.1)	
**FSS Scores**		
FSS total score		.27 (.06, .57)
Headache and gastrointestinal complaints		.17 (.00, .67)
Overtiredness, dizziness and musculoskeletal pain		.33 (.00, .67)

BMI = body mass index, hsCRP = high-sensitive C-reactive protein, HSV1 = herpes simplex virus type 1, HSV2 = herpes simplex virus type 2, EBV = Epstein barr virus, CMV = cytomegalovirus, HHV6 = human herpes virus type 6. FSS = functional somatic symptoms. AU = Arbitrary Units

^#^Median and percentiles were calculated in the group that was positive for antibodies against that virus

### Associations between antibody levels to herpes viruses and FSS scores

Results of our analyses on the association between the different herpes virus antibody levels in the seropositive groups and FSS scores are summarized in Table 2. Levels of antibodies to HSV1, HHV6, EBV and CMV were not associated with the FSS total score or the FSS score in both symptom clusters.

**Table 2:** Associations between the levels of viral antibodies and FSS.

		Total FSS scores	Headache and Gastrointestinal complaints	Overtiredness, Dizziness and Musculoskeletal pain
**HSV1 (AU)**	**B**	-.002	-.012	.006
	**95% CI**	-.046 to .045	-.075 to .057	-.041 to .052
	**p**	.93	.73	.80
**EBV (AU)**	**B**	-.028	-.003	-.034
	**95% CI**	-.133 to .068	-.151 to .143	-.127 to .059
	**p**	.56	.80	.49
**CMV (AU)**	**B**	-.004	.015	-.016
	**95% CI**	-.062 to .055	-.074 to .106	-.067 to .039
	**p**	.89	.72	.57
**HHV6 (AU)**	**B**	-.001	-.002	.002
	**95% CI**	-.011 to .011	-.017 to .013	-.005 to .009
	**p**	.92	.83	.52

HSV1 = herpes simplex virus type 1 antibody levels in the seropositive group (N = 301), EBV = Epstein barr virus antibody levels in the seropositive group (N = 305), CMV = cytomegalovirus antibody levels in the seropositive group (N = 306), HHV6 = human herpes virus 6 antibody levels in the seropositive group (N = 1117). AU = arbitrary units. All analyses were adjusted for sex, BMI, and affective problems and symptom scores in the other symptom group.

### Association between pathogen burden and FSS scores

Pathogen burden was not associated with FSS total score (B = -.005, 95%CI: -.022 to .014, p = .58), FSS scores in the symptom cluster of headache and gastrointestinal complaints (B = -.004, 95%CI: -.030 to .024, p = .76) or in the symptom cluster of overtiredness, dizziness and musculoskeletal pain (B = -.004, 95%CI: -.023 to .015, p = .70).

### Association between hsCRP as marker of inflammation and FSS scores

We found a significant association between hsCRP levels and the total FSS score (B = .02, 95%CI .004 to .028, p = .01). Regarding the different symptom clusters, we found a significant association between hsCRP and symptom scores in the symptom cluster of headache and gastrointestinal complaints (B = .02, 95%CI .002 to .039, p = .04), but not between hsCRP and symptom scores in the symptom cluster of overtiredness, dizziness and musculoskeletal pain (B = .006, 95%CI -.007 to .020, p = .39).

Using dichotomized hsCRP gave essentially the same results, with an association between elevated hsCRP and total FSS (B = .06, 95% CI: .005 to .113), p = .04), and an association between elevated hsCRP and symptom scores in the symptom cluster of headache and gastrointestinal complaints (B = .08, 95% CI: .005 to .158, p = .03) but no association between elevated hsCRP and symptom scores in the symptom cluster of overtiredness, dizziness and musculoskeletal pain (B = .02, 95% CI: -.034 to .075), p = .46).

### CRP as a mediator in the association between viral antibody levels and FSS

We found no association between the different levels of viral antibodies or pathogen burden and the FSS scores. We also found no association between the viral antibody levels or pathogen burden and hsCRP levels (Table 3). Therefore we did not proceed with mediation analysis for hsCRP. Using dichotomized hsCRP gave essentially the same results, with no associations between elevated hsCRP and antibody levels.

**Table 3:** Associations between level of viral antibodies and hsCRP levels.

	hsCRP levels (mg/l)
**HSV1 (AU)**	B = -.031, 95%CI: -.299 to .220, p = .80
**EBV (AU)**	B = -.347, 95%CI: -.730 to .110, p = .10
**CMV (AU)**	B = .192, 95%CI: -.198 to .656, p = .40
**HHV6 (AUl)**	B = .002, 95%CI: -.034 to .040, p = .92
**Pathogen Burden**	B = .156, 95%CI: -.062 to .391 p = .22

hsCRP = high sensitive C-reactive protein, HSV1 = herpes simplex virus type 1 antibody levels in the seropositive group (N = 287), EBV = Epstein barr virus antibody levels in the seropositive group (N = 296), CMV = cytomegalovirus antibody levels in the seropositive group (N = 298), HHV6 = human herpes virus 6 antibody levels in the seropositive group (N = 1082). Pathogen Burden = analysed in total population (N = 1220), AU = arbitrary units. All analyses were adjusted for sex, BMI and depressive symptoms.

## Discussion

Our study in adolescents showed no association between herpes virus infections or pathogen burden and FSS in general or specific FSS symptom groups. Thus, a precipitating effect of infection with herpes viruses on FSS was not found. A role for inflammatory processes in FSS development was supported by the significant association we found between hsCRP levels and FSS, especially in the symptom group of headache and gastrointestinal complaints.

A major strength of our study is the large sample size and the fact that we used a population cohort, which increases generalizability of our results. Another strength is the fact that we chose adolescents for our study. In adolescents, the influence of ageing-related factors on inflammatory processes is limited. An important limitation is the fact that we measured FSS with self-report questionnaires. Even though the adolescents were asked for complaints without a medical explanation, we cannot be sure that there was no physical cause for their complaints. The association in the FSS group of headache and gastrointestinal complaints with higher hsCRP values might suggest a physical explanation for the gastrointestinal complaints or the headache, such as a bowel infection or sinusitis. We did, however, exclude adolescents with hsCRP levels higher than 10 mg/L, making this explanation less likely. In large epidemiologic cohort studies, the assessment of FSS is mostly by self-report or parental report, without physical examinations [40,41]. This limitation is counterbalanced by the large number of adolescents that can be included in such cohort studies. The prevalence of the herpes viruses at age 16 in our population was low compared to findings in adolescents in age group 15–19 from other countries, with 70% seroprevalence for EBV in the USA [42], 1.6% for HSV2 in the USA, 39% for HSV1 in the USA [43], and 35% for CMV in Germany [44]. Especially EBV seropositivity was very low in our cohort. Factors associated with EBV seropositivity include ethnicity, crowdedness, household income, household education level and health insurance status [42]and differences in these factors between regions and nations might contribute to our low EBV seroprevalence. The rural character and relatively high proportion of Caucasian inhabitants in the region may contribute to the low prevalence of EBV. For our mediation analyses, we tested whether hsCRP was associated with FSS total scores and FSS in the different symptom clusters. We found an association of hsCRP and the total FSS scores, which was mainly explained by the symptom cluster of headache and gastro-intestinal complaints. However, it should be emphasized that this association would lose significance after correction for multiple testing. There is one previous study on hsCRP in FSS, which was performed in adults [19]. In this study, hsCRP was only associated with FSS in the symptom cluster of musculoskeletal pain; fatigue was not included in this symptom group. The symptom cluster of gastrointestinal complaints was not associated with hsCRP in that study. The most likely explanations for the inconsistent findings are differences in age groups and study designs, and varying symptoms in the clusters. Other studies focused on inflammation and pain, which is a common symptom in FSS. For example, it is found that cytokines excitate the neurotransmission of pain [45,46]. Furthermore, it was found that noxious stimuli increased the serum levels of IL-6 and that higher levels of catastrophizing thoughts during the admission of the pain stimuli gave greater pain-related increases in IL-6 [47]. The question remains what the cause of low grade inflammation might be. Our findings on the lack of association between hsCRP and antibody levels suggest that the herpes viruses were not causing systemic inflammation in our population. In a previous study in this cohort we focused on the effect of viruses and hsCRP on cognitive functioning. In this study we found an association for HSV-1 levels and cognitive functioning, but not for hsCRP, also suggesting that HSV-1 infection did not have its effect through systemic inflammation [48]. It is possible that other viral infections, that we did not measure, were the cause of systemic inflammation.

Another potential cause of systemic inflammation is psychological stress. There are several known mechanisms by which stress could influence the immune system, such as changes in HPA-axis responses, catecholamines, autonomous nervous system responses, epigenetic influences and health behaviors [49–52]. Some of these mechanisms have been studied in their relation to FSS in the TRAILS cohort. For example, FSS in the cluster of overtiredness, dizziness and musculoskeletal complaints were associated with low cortisol levels after awakening, whereas FSS in the cluster of gastrointestinal symptoms and headache were associated with low cortisol levels during a psychological stress task. These findings suggest that symptoms of headache and gastrointestinal complaints are associated with the experience of acute stress, whereas symptoms of overtiredness, dizziness and musculoskeletal pain are associated with chronic or recurrent stress [23]. Chronic and acute stress also differ in terms of inflammatory effects. Chronic stress is associated with circulating inflammatory parameters, whereas acute stress is related to inflammation in young adults but not in elderly people [49,50]. Thus, age differences could explain why we found an association between hsCRP and FSS in the group with headache and gastrointestinal complaints in our adolescents, whereas this was not found in an earlier study in adults. Another previous study in this cohort found that stressful life events were associated with elevated hsCRP. When subdomains of life events were studied, especially separation trauma and sexual abuse were associated with elevated hsCRP [53]. These findings were in line with findings from a recent meta-analysis that confirmed the association between childhood life events and inflammation later in life [54]. This could also be relevant for our study on the association between elevated hsCRP and FSS. It has consistently been found that the experience of childhood life events is associated with the presence of different types of FSS [55,56]. Earlier studies in this cohort also found an association between sexual abuse and FSS [57] and childhood adversities and FSS [58]. Thus, stress and life events might contribute to low grade systemic inflammation in FSS.

In conclusion, our study in adolescents does not confirm that FSS scores are associated with antibody levels against different herpes viruses or with pathogen burden. It does suggest that FSS scores are associated with hsCRP levels, but this finding needs to be replicated before drawing any conclusions. Obviously, infection or inflammation is not a standalone cause for the experience of FSS. Therefore it would be interesting to study the interdependence of different response mechanisms of the body, such as stress systems, hormone levels and inflammatory systems, and their effects on physical and psychological symptoms.

## Supporting information

S1 Dataset (XLSX)

## Abbreviations

FSS: functional somatic symptoms
hsCRP: high sensitive C-reactive protein
HSV1: Herpes Simplex Virus 1
HSV2: Herpes Simplex Virus 2
CMV: cytomegalovirus
EBV: Epstein Barr Virus
HHV6: human herpes virus 6

## References

[1] Henningsen P, Zipfel S, Herzog W. Management of functional somatic syndromes. Lancet 2007 3 17;369(9565):946–955. doi: 10.1016/S0140-6736(07)60159-7 17368156 10.1016/S0140-6736(07)60159-7

[2] Euteneuer F, Schwarz MJ, Hennings A, Riemer S, Stapf T, Selberdinger V, et al Psychobiological aspects of somatization syndromes: contributions of inflammatory cytokines and neopterin. Psychiatry Res 2012 1 30;195(1–2):60–65. doi: 10.1016/j.psychres.2011.07.032 21864915 10.1016/j.psychres.2011.07.032

[3] Dantzer R. Cytokine-induced sickness behavior: mechanisms and implications. Ann N Y Acad Sci 2001 3;933:222–234. 12000023 10.1111/j.1749-6632.2001.tb05827.x

[4] Klimas NG, Koneru AO. Chronic fatigue syndrome: inflammation, immune function, and neuroendocrine interactions. Curr Rheumatol Rep 2007 12;9(6):482–487. 18177602 10.1007/s11926-007-0078-y

[5] Sainz B, Loutsch JM, Marquart ME, Hill JM. Stress-associated immunomodulation and herpes simplex virus infections. Med Hypotheses 2001 3;56(3):348–356. doi: 10.1054/mehy.2000.1219 11359358 10.1054/mehy.2000.1219

[6] Cao Y, Zhang Y, Chang DF, Wang G, Zhang X. Psychosocial and immunological factors in neurasthenia. Psychosomatics 2009 Jan-Feb;50(1):24–29. doi: 10.1176/appi.psy.50.1.24 19213969 10.1176/appi.psy.50.1.24

[7] Halvorson HA, Schlett CD, Riddle MS. Postinfectious irritable bowel syndrome—a meta-analysis. Am J Gastroenterol 2006 8;101(8):1894–9; quiz 1942. doi: 10.1111/j.1572-0241.2006.00654.x 16928253 10.1111/j.1572-0241.2006.00654.x

[8] Bansal AS, Bradley AS, Bishop KN, Kiani-Alikhan S, Ford B. Chronic fatigue syndrome, the immune system and viral infection. Brain Behav Immun 2012 1;26(1):24–31. doi: 10.1016/j.bbi.2011.06.016 21756995 10.1016/j.bbi.2011.06.016

[9] Ablashi DV, Eastman HB, Owen CB, Roman MM, Friedman J, Zabriskie JB, et al Frequent HHV-6 reactivation in multiple sclerosis (MS) and chronic fatigue syndrome (CFS) patients. J Clin Virol 2000 5;16(3):179–191. 10738137 10.1016/s1386-6532(99)00079-7

[10] Lerner AM, Ariza ME, Williams M, Jason L, Beqaj S, Fitzgerald JT, et al Antibody to Epstein-Barr virus deoxyuridine triphosphate nucleotidohydrolase and deoxyribonucleotide polymerase in a chronic fatigue syndrome subset. PLoS One 2012;7(11):e47891 doi: 10.1371/journal.pone.0047891 23155374 10.1371/journal.pone.0047891 PMC3498272

[11] Cameron B, Flamand L, Juwana H, Middeldorp J, Naing Z, Rawlinson W, et al Serological and virological investigation of the role of the herpesviruses EBV, CMV and HHV-6 in post-infective fatigue syndrome. J Med Virol 2010 10;82(10):1684–1688. doi: 10.1002/jmv.21873 20827765 10.1002/jmv.21873

[12] Koelle DM, Barcy S, Huang ML, Ashley RL, Corey L, Zeh J, et al Markers of viral infection in monozygotic twins discordant for chronic fatigue syndrome. Clin Infect Dis 2002 9 1;35(5):518–525. doi: 10.1086/341774 12173124 10.1086/341774

[13] Reeves WC, Stamey FR, Black JB, Mawle AC, Stewart JA, Pellett PE. Human herpesviruses 6 and 7 in chronic fatigue syndrome: a case-control study. Clin Infect Dis 2000 7;31(1):48–52. doi: 10.1086/313908 10913395 10.1086/313908

[14] Buchwald D, Ashley RL, Pearlman T, Kith P, Komaroff AL. Viral serologies in patients with chronic fatigue and chronic fatigue syndrome. J Med Virol 1996 9;50(1):25–30. doi: 10.1002/(SICI)1096-9071(199609)50:1<25::AID-JMV6>3.0.CO;2-V 8890037 10.1002/(SICI)1096-9071(199609)50:1<25::AID-JMV6>3.0.CO;2-V

[15] Spiller R, Garsed K. Infection, inflammation, and the irritable bowel syndrome. Dig Liver Dis 2009 12;41(12):844–849. doi: 10.1016/j.dld.2009.07.007 19716778 10.1016/j.dld.2009.07.007

[16] Fouad YM, Makhlouf MM, Khalaf H, Mostafa Z, Abdel Raheem E, Meneasi W. Is irritable bowel syndrome associated with chronic hepatitis C? J Gastroenterol Hepatol 2010 7;25(7):1285–1288. doi: 10.1111/j.1440-1746.2010.06311.x 20594257 10.1111/j.1440-1746.2010.06311.x

[17] Moss-Morris R, Spence M. To "lump" or to "split" the functional somatic syndromes: can infectious and emotional risk factors differentiate between the onset of chronic fatigue syndrome and irritable bowel syndrome? Psychosom Med 2006 May-Jun;68(3):463–469. doi: 10.1097/01.psy.0000221384.07521.05 16738080 10.1097/01.psy.0000221384.07521.05

[18] Lacourt TE, Houtveen JH, Smeets HM, Lipovsky MM, van Doornen LJ. Infection Load as a Predisposing Factor for Somatoform Disorders: Evidence From a Dutch General Practice Registry. Psychosom Med 2013 10;75(8):759–764. doi: 10.1097/PSY.0b013e3182a3d91f 23960160 10.1097/PSY.0b013e3182a3d91f

[19] Tak LM, Bakker SJ, Slaets JP, Rosmalen JG. Is high-sensitive C-reactive protein a biomarker for functional somatic symptoms? A population-based study. Brain Behav Immun 2009 10;23(7):1014–1019. doi: 10.1016/j.bbi.2009.05.059 19501644 10.1016/j.bbi.2009.05.059

[20] Huisman M, Oldehinkel AJ, de Winter A, Minderaa RB, de Bildt A, Huizink AC, et al Cohort profile: the Dutch 'TRacking Adolescents' Individual Lives' Survey'; TRAILS. Int J Epidemiol 2008 12;37(6):1227–1235. doi: 10.1093/ije/dym273 18263649 10.1093/ije/dym273

[21] Achenbach TM, Dumenci L, Rescorla LA. DSM-oriented and empirically based approaches to constructing scales from the same item pools. J Clin Child Adolesc Psychol 2003 9;32(3):328–340. doi: 10.1207/S15374424JCCP3203_02 12881022 10.1207/S15374424JCCP3203_02

[22] de Groot A, Koot HM, Verhulst FC. Cross-cultural generalizability of the Youth Self-Report and Teacher's Report Form cross-informant syndromes. J Abnorm Child Psychol 1996 10;24(5):651–664. 8956089 10.1007/BF01670105

[23] Janssens KA, Oldehinkel AJ, Verhulst FC, Hunfeld JA, Ormel J, Rosmalen JG. Symptom-specific associations between low cortisol responses and functional somatic symptoms: The TRAILS study. Psychoneuroendocrinology 2011 7 29. 10.1016/j.psyneuen.2011.06.016 21803502

[24] Dickerson FB, Boronow JJ, Stallings C, Origoni AE, Ruslanova I, Yolken RH. Association of serum antibodies to herpes simplex virus 1 with cognitive deficits in individuals with schizophrenia. Arch Gen Psychiatry 2003 5;60(5):466–472. doi: 10.1001/archpsyc.60.5.466 12742867 10.1001/archpsyc.60.5.466

[25] Bergstrom T, Trybala E. Antigenic differences between HSV-1 and HSV-2 glycoproteins and their importance for type-specific serology. Intervirology 1996;39(3):176–184. 9058170 10.1159/000150493

[26] Buka SL, Tsuang MT, Torrey EF, Klebanoff MA, Bernstein D, Yolken RH. Maternal infections and subsequent psychosis among offspring. Arch Gen Psychiatry 2001 11;58(11):1032–1037. 11695949 10.1001/archpsyc.58.11.1032

[27] Ribes JA, Smith A, Hayes M, Baker DJ, Winters JL. Comparative performance of herpes simplex virus type 1-specific serologic assays from MRL and Meridian Diagnostics. J Clin Microbiol 2002 3;40(3):1071–1072. doi: 10.1128/JCM.40.3.1071-1072.2002 11880443 10.1128/JCM.40.3.1071-1072.2002 PMC120264

[28] Rifai N, Ridker PM. High-sensitivity C-reactive protein: a novel and promising marker of coronary heart disease. Clin Chem 2001 3;47(3):403–411. 11238289

[29] Danner M, Kasl S, Abramson JL, Vaccarino V. Association between depression and elevated C-reactive protein. Psychosomatic medicine 2003;65(3):347–356. 12764206 10.1097/01.psy.0000041542.29808.01

[30] Ford DE, Erlinger TP. Depression and C-reactive protein in US adults: data from the Third National Health and Nutrition Examination Survey. Archives of Internal Medicine 2004;10;164(9):1010–1014. 10.1001/archinte.164.9.1010 15136311

[31] Karlovic D, Serretti A, Vrkic N, Martinac M, Marcinko D. Serum concentrations of CRP, IL-6, TNF-alpha and cortisol in major depressive disorder with melancholic or atypical features. Psychiatry Res 2012 6 30;198(1):74–80. doi: 10.1016/j.psychres.2011.12.007 22386567 10.1016/j.psychres.2011.12.007

[32] Wang G, Christoffel KK, Brickman WJ, Hong X, Arguelles L, Zhang S, et al C-reactive protein in adolescent twins: patterns and relationship to adiposity. J Clin Endocrinol Metab 2011 10;96(10):3226–3233. doi: 10.1210/jc.2011-0590 21832113 10.1210/jc.2011-0590 PMC3200237

[33] Sbarouni E, Georgiadou P, Voudris V. Gender-specific differences in biomarkers responses to acute coronary syndromes and revascularization procedures. Biomarkers 2011 9;16(6):457–465. doi: 10.3109/1354750X.2011.576431 21851313 10.3109/1354750X.2011.576431

[34] Kato K, Sullivan PF, Pedersen NL. Latent class analysis of functional somatic symptoms in a population-based sample of twins. J Psychosom Res 2010 May;68(5):447–453. doi: 10.1016/j.jpsychores.2010.01.010 20403503 10.1016/j.jpsychores.2010.01.010 PMC2858068

[35] Paulis WD, Silva S, Koes BW, van Middelkoop M. Overweight and obesity are associated with musculoskeletal complaints as early as childhood: a systematic review. Obes Rev 2014 1;15(1):52–67. doi: 10.1111/obr.12067 23941399 10.1111/obr.12067

[36] Howren MB, Lamkin DM, Suls J. Associations of depression with C-reactive protein, IL-1, and IL-6: a meta-analysis. Psychosom Med 2009 2;71(2):171–186. doi: 10.1097/PSY.0b013e3181907c1b 19188531 10.1097/PSY.0b013e3181907c1b

[37] Campo JV. Annual Research Review: Functional somatic symptoms and associated anxiety and depression—developmental psychopathology in pediatric practice. J Child Psychol Psychiatry 2012 5;53(5):575–592. doi: 10.1111/j.1469-7610.2012.02535.x 22404290 10.1111/j.1469-7610.2012.02535.x

[38] Janssens KA, Rosmalen JG, Ormel J, van Oort FV, Oldehinkel AJ. Anxiety and depression are risk factors rather than consequences of functional somatic symptoms in a general population of adolescents: the TRAILS study. J Child Psychol Psychiatry 2010 3;51(3):304–312. doi: 10.1111/j.1469-7610.2009.02174.x 19788552 10.1111/j.1469-7610.2009.02174.x

[39] Wright DB, London K, Field AP. Using Bootstrap Estimation and the Plug-in Principle for Clinical Psychology Data. Journal of Experimental Psychopathology 2011;2:252–270.

[40] Rask CU, Ornbol E, Olsen EM, Fink P, Skovgaard AM. Infant behaviors are predictive of functional somatic symptoms at ages 5–7 years: results from the Copenhagen Child Cohort CCC2000. J Pediatr 2013 2;162(2):335–342. doi: 10.1016/j.jpeds.2012.08.001 23026486 10.1016/j.jpeds.2012.08.001

[41] Ramchandani PG, Hotopf M, Sandhu B, Stein A, ALSPAC Study Team. The epidemiology of recurrent abdominal pain from 2 to 6 years of age: results of a large, population-based study. Pediatrics 2005 7;116(1):46–50. doi: 10.1542/peds.2004-1854 15995029 10.1542/peds.2004-1854

[42] Balfour HH Jr, Sifakis F, Sliman JA, Knight JA, Schmeling DO, Thomas W. Age-specific prevalence of Epstein-Barr virus infection among individuals aged 6–19 years in the United States and factors affecting its acquisition. J Infect Dis 2013 10 15;208(8):1286–1293. doi: 10.1093/infdis/jit321 23868878 10.1093/infdis/jit321

[43] Xu F, Sternberg MR, Kottiri BJ, McQuillan GM, Lee FK, Nahmias AJ, et al Trends in herpes simplex virus type 1 and type 2 seroprevalence in the United States. JAMA 2006 8 23;296(8):964–973. doi: 10.1001/jama.296.8.964 16926356 10.1001/jama.296.8.964

[44] Enders G, Daiminger A, Lindemann L, Knotek F, Bader U, Exler S, et al Cytomegalovirus (CMV) seroprevalence in pregnant women, bone marrow donors and adolescents in Germany, 1996–2010. Med Microbiol Immunol 2012 8;201(3):303–309. doi: 10.1007/s00430-012-0232-7 22398714 10.1007/s00430-012-0232-7

[45] Binshtok AM, Wang H, Zimmermann K, Amaya F, Vardeh D, Shi L, et al Nociceptors are interleukin-1beta sensors. J Neurosci 2008 12 24;28(52):14062–14073. doi: 10.1523/JNEUROSCI.3795-08.2008 19109489 10.1523/JNEUROSCI.3795-08.2008 PMC2690713

[46] Uceyler N, Schafers M, Sommer C. Mode of action of cytokines on nociceptive neurons. Exp Brain Res 2009 6;196(1):67–78. doi: 10.1007/s00221-009-1755-z 19290516 10.1007/s00221-009-1755-z

[47] Edwards RR, Kronfli T, Haythornthwaite JA, Smith MT, McGuire L, Page GG. Association of catastrophizing with interleukin-6 responses to acute pain. Pain 2008 11 15;140(1):135–144. doi: 10.1016/j.pain.2008.07.024 18778895 10.1016/j.pain.2008.07.024 PMC2659503

[48] Jonker I, Klein HC, Duivis HE, Yolken RH, Rosmalen JG, Schoevers RA. Association between Exposure to HSV1 and Cognitive Functioning in a General Population of Adolescents. The TRAILS Study. PLoS One 2014 7 1;9(7):e101549 doi: 10.1371/journal.pone.0101549 24983885 10.1371/journal.pone.0101549 PMC4077793

[49] Rohleder N. Acute and chronic stress induced changes in sensitivity of peripheral inflammatory pathways to the signals of multiple stress systems—2011 Curt Richter Award Winner. Psychoneuroendocrinology 2012 3;37(3):307–316. doi: 10.1016/j.psyneuen.2011.12.015 22226321 10.1016/j.psyneuen.2011.12.015

[50] Rohleder N, Wolf JM, Kirschbaum C. Glucocorticoid sensitivity in humans-interindividual differences and acute stress effects. Stress 2003 9;6(3):207–222. doi: 10.1080/1025389031000153658 13129814 10.1080/1025389031000153658

[51] Dhabhar FS. Effects of stress on immune function: the good, the bad, and the beautiful. Immunol Res 2014 5;58(2–3):193–210. doi: 10.1007/s12026-014-8517-0 24798553 10.1007/s12026-014-8517-0

[52] Acabchuk RL, Kamath J, Salamone JD, Johnson BT. Stress and chronic illness: The inflammatory pathway. Soc Sci Med 2017 7;185:166–170. doi: 10.1016/j.socscimed.2017.04.039 28552293 10.1016/j.socscimed.2017.04.039 PMC8570552

[53] Jonker I, Rosmalen JGM, Schoevers RA. Childhood life events, immune activation and the development of mood and anxiety disorders: the TRAILS study. Transl Psychiatry 2017 5 2;7(5):e1112 doi: 10.1038/tp.2017.62 28463238 10.1038/tp.2017.62 PMC5534944

[54] Baumeister D, Akhtar R, Ciufolini S, Pariante CM, Mondelli V. Childhood trauma and adulthood inflammation: a meta-analysis of peripheral C-reactive protein, interleukin-6 and tumour necrosis factor-alpha. Mol Psychiatry 2015 6 2. 10.1038/mp.2015.67 PMC4564950 26033244

[55] Karatzias T, Howard R, Power K, Socherel F, Heath C, Livingstone A. Organic vs. functional neurological disorders: The role of childhood psychological trauma. Child Abuse Negl 2017 1;63:1–6. doi: 10.1016/j.chiabu.2016.11.011 27886517 10.1016/j.chiabu.2016.11.011

[56] Roelofs K, Spinhoven P. Trauma and medically unexplained symptoms towards an integration of cognitive and neuro-biological accounts. Clin Psychol Rev 2007 10;27(7):798–820. doi: 10.1016/j.cpr.2007.07.004 17728032 10.1016/j.cpr.2007.07.004

[57] Bonvanie IJ, van Gils A, Janssens KA, Rosmalen JG. Sexual abuse predicts functional somatic symptoms: an adolescent population study. Child Abuse Negl 2015 8;46:1–7. doi: 10.1016/j.chiabu.2015.06.001 26142915 10.1016/j.chiabu.2015.06.001

[58] Bonvanie IJ, Janssens KA, Rosmalen JG, Oldehinkel AJ. Life events and functional somatic symptoms: A population study in older adolescents. Br J Psychol 2017 5;108(2):318–333. doi: 10.1111/bjop.12198 27221984 10.1111/bjop.12198

